# Actin polymerization is reduced in the anterior cingulate cortex of elderly patients with schizophrenia

**DOI:** 10.1038/s41398-017-0045-y

**Published:** 2017-12-11

**Authors:** Hriday P. Bhambhvani, Toni M. Mueller, Micah S. Simmons, James H. Meador-Woodruff

**Affiliations:** Department of Psychiatry and Behavioral Neurobiology, University of Alabama at Birmingham, 1719 6th Avenue South, CIRC 593A, Birmingham, AL 35294 USA

## Abstract

Recent reports suggest abnormalities in the regulation of actin cytoskeletal dynamics in schizophrenia, despite consistent evidence for normal actin expression. We hypothesized that this may be explained by changes in the polymerization state of actin, rather than in total actin expression. To test this, we prepared filamentous actin (F-actin, polymeric) and globular actin (G-actin, monomeric) fractions from postmortem anterior cingulate cortex from 16 patients with schizophrenia and 14 comparison subjects. Additionally, binding of fluorescently-labeled phalloidin, a selectively F-actin-binding peptide, was measured in unfractionated samples from the same subjects. Western blot analysis of fractions revealed decreased F-actin, increased G-actin, and decreased ratios of F-actin/total actin and F-actin/G-actin in schizophrenia. Decreased phalloidin binding to F-actin in parallel experiments in the same subjects independently supports these findings. These results suggest a novel aspect of schizophrenia pathophysiology and are consistent with previous evidence of reduced dendritic spine density and altered synaptic plasticity in schizophrenia, both of which have been linked to cytoskeletal abnormalities.

## Introduction

While the etiology of schizophrenia is not established, a consistent finding in schizophrenia brain is reduced dendritic spine density and altered spine morphology^1–4^. The cause of these dendritic spine abnormalities is unknown. One hypothesis implicates dysfunction of the actin cytoskeleton, the stabilization of which is essential for maintaining dendritic spine morphology and density^5^. Consistent with this hypothesis, many proteins crucial to stabilization of the actin cytoskeleton have been shown to be abnormally expressed in schizophrenia, including reelin, fragile X mental retardation protein, disrupted in schizophrenia-1 (DISC-1), cell division cycle protein 42 (CDC42), and Duo^6–9^. These abnormalities are intriguing, however, given multiple reports of normal actin protein expression in schizophrenia brain^10, 11^. The discrepancy may be explained by altered actin polymerization, abnormal binding of actin to cytoskeletal substrates such as cofilin and N-WASP, or abnormal turnover and restructuring of the actin cytoskeleton. Actin remodeling is known to drive both the formation and loss of dendritic spines, as well as regulate their morphological plasticity^12, 13^. Underlying this rearrangement of actin is the dynamic polymerization of actin from its monomeric form (G-actin) to a filamentous polymer (F-actin), both of which are present in spines and affect spine morphology^14^. Interestingly, it has been hypothesized that diminished cortical dendritic spine density and the resulting reduction in excitatory projections to the ventral mesencephalon, the location of the dopamine cells that project to striatum and limbic areas, gives rise to a hyperdopaminergic state in schizophrenia^15^. Indeed, reduction of actin polymerization via deletion of the actin related protein-2/3 (Arp2/3) complex in mice induced a loss of dendritic spines and elevated striatal dopaminergic neurotransmission^16^.

Proteins involved in pathways regulating actin polymerization have been found to be abnormally expressed in schizophrenia. The Arp2/3 complex is a chief initiator of actin polymerization and regulates the formation of branched actin networks^17^. Previously, we reported reduced protein expression of cortactin, a nucleation promoting factor (NPF) and activator of the Arp2/3 complex^18^. Additionally, reduced gene expression of cortactin, CYFIP1 (NPF), N-WASP (NPF and activator of the Arp2/3 complex), Arp2, and Arp3 was recently reported^19^. Altogether, these data suggest impaired actin polymerization in schizophrenia. We hypothesized that, although total actin expression is unchanged in schizophrenia, the polymerization of actin may be decreased. To test this hypothesis, we applied an F-actin/G-actin fractionation technique used frequently in cell culture studies to postmortem human brain. F-actin and G-actin enriched fractions of anterior cingulate cortex from schizophrenia and comparison subjects were probed for actin using western blot analysis to determine actin polymerization status. In the same subjects, fluorescently-labeled phalloidin, a selectively F-actin-binding peptide, was used as an independent measure of F-actin.

## Methods

### Subjects acquisition and tissue processing

Samples from the full thickness of the gray matter of anterior cingulate cortex (ACC, BA24/32) of 16 schizophrenia and 14 comparison subjects (Table 1) were obtained from the Mount Sinai Medical Center brain collection, as previously described^20^. The diagnosis of schizophrenia was determined by two independent clinicians using DSM-III-R criteria^21^. All patients had a history of psychotic symptoms prior to the age of 40 and at least 10 years of hospitalization. Patients were recruited prospectively and underwent extensive antemortem clinical assessment. Subject medical history was assessed for alcoholism, substance abuse, death by suicide, coma for more than 6 h before death, or evidence of neurodegenerative disorders, and subjects were excluded if any of these were present. Comparison subjects were assessed similarly and confirmed to be without neuropathological evidence of neurodegenerative disorders and history of psychiatric illness or substance abuse. Each sample was snap frozen, pulverized with small amounts of liquid nitrogen, and stored at −80 °C until use.

**Table 1:** Summary of subject demographics

	Comparison	Schizophrenia	p
*n*	14	16	
Age	79.4 ± 9.3	75.8 ± 11.9	0.36
Sex	4 M/10 F	11 M/5 F	0.03
PMI (hours)	10.0 ± 7.3	11.4 ± 4.4	0.54
Tissue pH	6.3 ± 0.2	6.4 ± 0.3	0.28
On/Off Rx	0/14	11/5	

Values are expressed as means ± standard deviation. Off Rx indicates patients who had not received antipsychotic medications for 6 weeks or more prior to time of death

*M* male, *F* female, *PMI* postmortem interval, *Rx* antipsychotic medication

### Antipsychotic drug-treated rats

The Institutional Animal Care and Use Committee of the University of Alabama at Birmingham approved all procedures using animals. Male Sprague Dawley rats (Charles River, Wilmington, MA) were intramuscularly injected every three weeks with either haloperidol decanoate in sesame oil (28.5 mg/kg, *N* = 10) or sesame oil only (*N* = 10) for a total of 12 injections over 9 months. The dose and duration of treatment were chosen to model chronic antipsychotic treatment in humans^9, 22^. The animals were decapitated and brains immediately harvested, dissected, then stored at −80 °C. Samples of rat cortex were used in this study.

### F-actin and G-actin fractionation

Tissue from both human and rat samples was homogenized in F-actin stabilization buffer [0.1 M PIPES, pH 6.9, 30% glycerol, 5% dimethyl sulfoxide, 1 mM MgSO_4_, 1 mM EGTA, 1% TritonX-100, 1 mM adenosine triphosphate, and protease inhibitor (Complete Mini, Roche Diagnostics, Manheim, Germany)] using glass pestle tissue grinders and the protein concentration of the resulting homogenates was measured by BCA protein assay (Thermo Scientific, Rockford, Illinois). Samples were incubated at 37 °C for 10 min and subsequently centrifuged at 2000 rpm for 5 min. The resulting supernatant was collected and centrifuged at 134,000 x *g* at 37 °C for 1 h in an ultracentrifuge (SW60Ti rotor, Beckman Coulter, Brea, CA) to partition G-actin (supernatant) from F-actin (pellet) fractions. The pellet was resuspended in 8 M urea, incubated on ice for 1 h, and vortexed every 15 min to depolymerize F-actin.

This protocol was adapted from and has been used extensively in cell culture studies^23–29^. To our knowledge, there are no existing reports of using this protocol in postmortem brain. To validate enrichment of F and G-actin from brain tissue, samples from two human comparison subjects were fractionated in triplicate. The resulting fractions were treated with Phalloidin-iFluor 488 Reagent (Abcam, Cambridge, United Kingdom) at 1:500 dilution for 1 h at room temperature. Fluorescence was measured using a Synergy HT Microplate Reader (BioTek, Winooski, VT) and the three measures for each subject were averaged. Homogenate without phalloidin treatment was measured as a negative control.

### Western blot analysis

Samples were reconstituted in 12.5 µl of ultrapure water (Milli-Q A10, Millipore, Billerica, MA) and 12.5 µl 6x reducing buffer (6x solution: 5% SDS, 15% β-mercaptoethanol, 0.0018% bromophenol blue and 36% glycerol in 170 mM Tris-HCl pH 6.8) and heated at 70 °C for 10 min. Samples were processed by sodium dodecyl sulfate polyacrylamide gel electrophoresis using Invitrogen (Carlsbad, CA) Bolt 4–12% Bis-Tris Plus gels, electrophoresed using a Novex Bolt Mini system (Life Technologies, Grand Island, NY), and transferred onto nitrocellulose membranes (Bio-Rad, Hercules, CA) using a semi-dry transfer apparatus (Bio-Rad). Membranes were blocked with Odyssey blocking buffer (LI-COR Biosceinces, Lincoln, NE) for 1 h at room temperature prior to incubation with anti-actin monoclonal antibody (MAB1501, Millipore) for 16 h at 4 °C. Membranes were washed with phosphate-buffered saline (PBS) containing 0.1% Tween-20 (PBST), and then probed with IR-Dye labeled secondary antibodies (LI-COR Biosciences). Membranes were again washed with PBST and placed in water prior to being imaged with a LI-COR Odyssey scanner. Protein bands of interest were measured using Image Studio 5.2 analytical software (LI-COR Biosciences).

The near-infrared fluorescence value for each target was normalized to the intralane value of β-tubulin, and technical replicates were averaged. β-tubulin was chosen as a loading control because it is ubiquitously expressed in human brain and was found to partition in both fractions. We found no difference in β-tubulin expression between schizophrenia and comparison groups in the G-actin fraction, F-actin fraction, or total (summed expression from G-actin and F-actin fractions), the last of which is consistent with prior reports^10, 30^.

### Phalloidin-binding assay

Tissue homogenized in F-actin stabilization buffer, as described above, containing 25 µg of protein was obtained from each subject. Triplicate samples from each subject were loaded in a 96-well plate (ThermoFisher) and treated with Phalloidin-iFluor 488 Reagent (Abcam) at 1:500 dilution for 1 h at room temperature. On the same plate, an equal volume of 1:500 phalloidin was placed in three wells without any sample for background measurement. Fluorescence was measured using a Synergy HT Microplate Reader (BioTek, Winooski, VT) and the average of three wells for each subject was calculated. To correct for background fluorescence in the assay, the average fluorescence of negative control wells was subtracted from each measure prior to analysis.

### Data analysis

Raw total actin (ACT_raw_) was calculated as the sum of average F-actin and average G-actin expression for each subject. The standard for data analysis using this fractionation methodology is to evaluate the ratios of F-actin/G-actin, F-actin/ACT_raw_, and G-actin/ACT_raw_^23–29^. These ratios represent the amount of F-actin and G-actin relative to total actin and to each other. As an additional control, we also normalized F-actin and G-actin bands to intralane β-tubulin from each sample.

Data were analyzed using Prism 6.07 software (GraphPad Software Inc., La Jolla, CA). The investigator who executed experimental protocols was blind to subject diagnosis until study completion. The D’Agostino-Pearson omnibus test was used to assess the normality of the distribution. Dependent variables not normally distributed were log transformed and verified to be normally distributed following transformation. Between group differences were assessed by two-tailed, unpaired Student’s *t*-tests. For significantly different dependent measures, post hoc assessments were performed using linear regression to identify potential relationships between protein expression and subject age, tissue pH, and postmortem interval. None of these factors demonstrated any significant association with our dependent measures. Due to the difference in sex distribution between diagnostic groups (*χ* ^2^ = 4.82, *p* = 0.03), post hoc two-way analysis of variance (ANOVA) was performed on significantly different dependent measures to assess potential sex differences. One-way ANOVA was used to assess potential differences related to antipsychotic treatment at the time of death. For all statistical tests, *α* = 0.05.

## Results

### Validation of F- and G-actin fractionation

The fractionation technique employed has been used frequently in cell culture studies^23–29^, but has not yet been validated or optimized for use with samples from postmortem human brain. We measured phalloidin binding in prepared fractions to validate the enrichment of filamentous actin in the F-actin fraction, and to confirm depletion of F-actin binding in the G-actin fraction. Our results indicate substantial binding of phalloidin in the F-actin fraction, consistent with enrichment of polymeric F-actin in this fraction. As expected, we found no phalloidin binding in the G-actin fraction. This finding reflects a complete depletion of F-actin from the G-actin fraction in fractionated postmortem brain samples (Table 2).

**Table 2:** F-actin and G-actin fractionation validation

	Subject 1	Subject 2
Phalloidin-treated F-Actin Fraction	28 ± 2	30 ± 2
Phalloidin-treated G-actin fraction	7 ± 1	8 ± 1
Untreated homogenate control	7 ± 1	8 ± 1

Values are relative fluorescence (arbitrary units) and reported as means ± standard deviation of three technical replicates from each subject for each condition

### Decreased F-actin and increased G-actin in schizophrenia

**Fig. 1:**
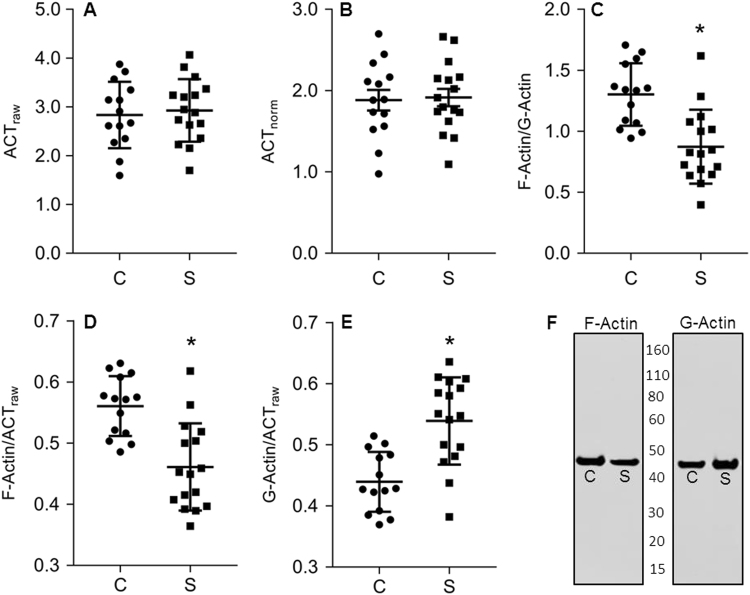
Actin polymerization is reduced in schizophrenia, while total actin expression remains unchanged **a** Raw total actin (ACT_raw_), the sum of the averaged F- and G-actin bands, is unchanged in schizophrenia. **b** β-tubulin-normalized total actin (ACT_norm_) is also unchanged in schizophrenia. **c** In schizophrenia, the ratio of F- to G-actin is decreased. **d** The ratio of F-actin to ACT_raw_ is decreased, while **e** the ratio of G-actin to ACT_raw_ increased. **f** Representative immunoblots of F-actin and G-actin. *p<0.001

We found that both raw total actin (ACT_raw_) and β-tubulin-normalized total actin (ACT_norm_) are unchanged between diagnostic groups (Fig. 1a, b). The ratio of F/G-actin was decreased by 33% in schizophrenia (Fig. 1c). F-actin/ACT_raw_, a measure of the extent of actin polymerization, was decreased by 18% in schizophrenia (Fig. 1d), and β-tubulin-normalized F-actin was decreased by 17% in schizophrenia (Table 3). G-actin/ACT_raw_ was increased by 23% (Fig. 1e), and β-tubulin-normalized G-actin expression was increased by 25% in schizophrenia relative to comparison subjects (Table 3). When the same dependent measures were assessed in rats chronically treated with an antipsychotic drug, none were found to be significantly different between rats treated with haloperidol and vehicle-treated controls (Table 4).

**Table 3:** Total actin, F-actin, G-actin, and phalloidin-binding levels in schizophrenia and comparison subjects

	Comparison	Schizophrenia	Test statistic	p
ACT_raw_	2.84 ± 0.68	2.93 ± 0.64	t(28) = 0.38	
F-actin/ACT_raw_	0.56 ± 0.05	0.46 ± 0.07	t(28) = 4.39	0.0001
G-actin/ACT_raw_	0.44 ± 0.05	0.54 ± 0.07	t(28) = 4.39	0.0001
F-actin/G-actin	1.30 ± 0.26	0.89 ± 0.07	t(28) = 4.37	0.0002
ACT_norm_	1.88 ± 0.47	1.92 ± 0.43	t(28) = 0.20	
F-actin/ β-tubulin	1.05 ± 0.25	0.87 ± 0.21	t(28) = 2.11	0.0439
G-actin/ β-tubulin	0.83 ± 0.24	1.04 ± 0.28	t(28) = 2.19	0.0373
Phalloidin (F-actin)	67.95 ± 16.12	47.65 ± 15.15	t(28) = 3.56	0.0014

Total raw actin (ACT_raw_), β-tubulin-normalized total actin (ACT_norm_), and reported ratios are calculated as described in Methods-Data Analysis. Phalloidin (F-actin) is calculated as the average fluorescence value (arbitratry units) for each subject less the average fluorescence of negative control samples containing only phalloidin. Data are reported as means ± standard deviation

**Table 4:** Total actin, F-actin, G-actin, and phalloidin-binding levels in haloperidol and vehicle-treated rats

	Control	Haloperidol	Test statistic
ACT_raw_	2.97 ± 0.03	3.01 ± 0.03	t(18) = 0.37
F-actin/ACT_raw_	0.51 ± 0.01	0.50 ± 0.02	t(18) = 1.22
G-actin/ACT_raw_	0.49 ± 0.01	0.50 ± 0.02	t(18) = 0.70
F-actin/G-actin	0.99 ± 0.06	0.98 ± 0.04	t(18) = 0.31
ACT_norm_	1.97 ± 0.06	1.99 ± 0.06	t(18) = 0.70
F-actin/ β-tubulin	0.98 ± 0.03	1.00 ± 0.04	t(18) = 1.63
G-actin/ β-tubulin	0.99 ± 0.06	0.98 ± 0.04	t(18) = 0.31
Phalloidin (F-actin)	58.27 ± 10.61	58.13 ± 8.93	t(18) = 0.03

Total raw actin (ACT_raw_), β-tubulin-normalized total actin (ACT_norm_), and reported ratios are calculated as described in Methods-Data Analysis. Phalloidin (F-actin) is calculated as the average fluorescence value (arbitratry units) for each subject less the average fluorescence of negative control samples containing only phalloidin. Data are reported as means ± standard deviation; no variables were significantly different between treatment conditions

### Phalloidin binding is reduced in schizophrenia

Phalloidin binding, an accepted measure of F-actin protein expression, was decreased by 30% in schizophrenia relative to non-psychiatrically ill comparison subjects (Table 3). Phalloidin binding was unchanged in antipsychotic-treated rats (Table 4).

## Discussion

In this study, we measured the extent of actin polymerization in the anterior cingulate cortex of schizophrenia and comparison subjects using both an F-actin/G-actin fractionation technique and the selectively F-actin-binding protein phalloidin. Western blot analysis of F-actin and G-actin fractions revealed decreased expression of F-actin when assessed independently and relative to total actin expression. Treatment of subjects with fluorescently-labeled phalloidin also independently revealed decreased expression of F-actin. In conjunction with a reduced F/G-actin ratio, these findings are consistent with our hypothesis that actin polymerization is impaired in schizophrenia.

Actin is a crucial component of cellular scaffolding responsible for establishing and maintaining cell morphology, and actin dynamics underlie core cellular processes including cell motility and intracellular protein trafficking^31^. In developing neurons, actin remodeling is vital to synaptogenesis and in the formation, extension, and development of neurites. In mature neurons, actin is the most abundant cytoskeletal protein at synapses and is present at both pre- and postsynaptic terminals. Modulation of actin dynamics is necessary for the cytoarchitectural changes that accompany synaptic plasticity^32–35^.

Actin polymerization is stimulated by nucleating factors, the most prominent of which is the Arp2/3 complex^36^. Interestingly, Arp2/3 knock-out mice exhibit many hallmark neurobiological traits of schizophrenia, including decreased spine formation, enhanced excitation of cortical neurons, and abnormal striatal output, suggesting dysfunction of the complex in this illness^16^. Knockdown of cortactin and N-WASP, NPFs, and activators of the Arp2/3 complex, results in decreased dendritic spine density in hippocampal neurons^37, 38^. Multiple lines of evidence suggest reduced activity of the Arp2/3 complex in schizophrenia^18, 19^, which could in turn diminish actin polymerization, as our current findings suggest.

Converging evidence has implicated synaptic dysfunction and reduced dendritic spine density as key components in the pathophysiology of schizophrenia^1–5, 39^. It is thought that enlargement of dendritic spines is facilitated by an increase in the F-actin/G-actin ratio, while reduced spine size is associated with a decreased F-actin/G-actin ratio^40, 41^. Reduced actin polymerization indicates impaired actin nucleation and/or cross-linking of F-actin filaments, processes which are critical for the activity-dependent structural plasticity of spines, which can contribute to decreased spine stability and ultimately spine loss^13^. Decreased cortical dendritic spine density has been hypothesized to be associated with hyperdopaminergia in schizophrenia^19^. Our findings of decreased F-actin and increased G-actin in schizophrenia (Fig. 1, Table 3) are consistent with this model, and suggest a mechanism that contributes to the reduced dendritic spine density and spine length in the illness. Enhancing actin polymerization might increase the density and length of dendritic spines, and thus, targeted modulation of actin polymerization in brain may be an exciting avenue for further exploration.

There are some limitations to this study in postmortem human brain. The patients studied were elderly and in late stages of the illness, and these findings may not generalize to younger patients. Another potential limitation of the current study is that the ratio of male:female subjects was different between diagnostic groups. To account for this, we conducted post hoc two-way ANOVA for all significantly different dependent measures using sex and diagnosis as the independent variables. We did not identify any effect of sex on protein expression.

Potential effects of chronic antipsychotic treatment on protein expression are also a concern in schizophrenia studies. To address the possibility that changes in actin polymerization status may be due to schizophrenia subjects receiving chronic antipsychotic treatment, we conducted parallel experiments in rats chronically treated with haloperidol. Protein expression of F-actin, G-actin, ACT_raw_, ACT_norm_, and ratios of actin forms, as well as phalloidin binding, were not different between haloperidol and vehicle-treated rats (Table 4). We also performed post hoc statistical analyses for all significantly different dependent measures between schizophrenia subjects who received antipsychotic treatment within 6 weeks of death vs. those who had been off medication for at least 6 weeks prior to death. No medication effects were identified via post hoc statistical analyses nor by alterations in an antipsychotic-treated rat model. Thus, it is unlikely that the abnormalities reported here are due to chronic medication effects, and these findings are more likely associated with the illness itself.

We identified reduced protein expression of F-actin in schizophrenia using converging techniques, as well as increased G-actin expression and reduced F/G-actin ratio. These findings are consistent with decreased actin polymerization, and suggest a mechanistic explanation for altered dendritic spine morphology and abundance observed in schizophrenia. Dendritic spines are the prime recipients of excitatory synaptic input and decreased dendritic spine density is known to impair synaptic plasticity, which may contribute to cognitive dysfunction associated with schizophrenia. These data implicate decreased actin polymerization as a potential substrate of the pathophysiology of this illness and may suggest actin remodeling as a potential target for novel treatments.
